# SNARE protein SEC22B regulates early embryonic development

**DOI:** 10.1038/s41598-019-46536-7

**Published:** 2019-08-07

**Authors:** Shin-Rong J. Wu, Rami Khoriaty, Stephanie H. Kim, K. Sue O’Shea, Guojing Zhu, Mark Hoenerhoff, Cynthia Zajac, Katherine Oravecz-Wilson, Tomomi Toubai, Yaping Sun, David Ginsburg, Pavan Reddy

**Affiliations:** 1 Program in Immunology, University of Michigan Medical School, Ann Arbor, USA; 2 Medical Scientist Training Program, University of Michigan Medical School, Ann Arbor, USA; 3 Department of Internal Medicine, Michigan Medicine, Ann Arbor, USA; 4 Department of Cellular and Developmental Biology, University of Michigan Medical School, Ann Arbor, USA; 5 Life Sciences Institute, University of Michigan, Ann Arbor, USA; 6 Unit for Laboratory Animal Medicine, University of Michigan, Ann Arbor, USA; 7 Department of Human Genetics, University of Michigan Medical School, Ann Arbor, USA; 8 Howard Hughes Medical Institute, University of Michigan, Ann Arbor, USA

**Keywords:** Embryology, Development

## Abstract

The highly conserved SNARE protein SEC22B mediates diverse and critical functions, including phagocytosis, cell growth, autophagy, and protein secretion. However, these characterizations have thus far been limited to *in vitro* work. Here, we expand our understanding of the role *Sec22b* plays *in vivo*. We utilized *Cre-Lox* mice to delete *Sec22b* in three tissue compartments. With a germline deletion of *Sec22b*, we observed embryonic death at E8.5. Hematopoietic/endothelial cell deletion of *Sec22b* also resulted in *in utero* death. Notably, mice with *Sec22b* deletion in CD11c-expressing cells of the hematopoietic system survive to adulthood. These data demonstrate *Sec22b* contributes to early embryogenesis through activity both in hematopoietic/endothelial tissues as well as in other tissues yet to be defined.

## Introduction

Intracellular trafficking plays a critical role in cellular biology, regulating the distribution and organization of secretory proteins. One protein class which helps mediate this complex choreography is the SNAREs (soluble NSF attachment protein receptor). Partner SNAREs bind and mediate the fusion of two membranes by physically bringing the membranes sufficiently close to fuse^1^. SEC22B is an endoplasmic reticulum (ER)-SNARE which localizes to the ER and the ER-Golgi intermediate compartment^2^. It functions as a vesicular-SNARE^3,4^ and an R-SNARE engaged in antero- and retrograde ER-Golgi transport^5,6^. Its known interacting partners are varied. It forms a classic four-helix SNARE complex with Qa-SNARE syntaxin 18, Qb-SNARE BNIP1, and Qc-SNARE p31/Use1 at the ER membrane^7^. SEC22B is also known to interact with plasma membrane/Qa-SNAREs syntaxin 1^8^, syntaxin 4^2^ and syntaxin 5^4^, suggesting it also functions at the interface between the plasma membrane and the ER membrane.

At the cellular level, in addition to its role in ER-Golgi trafficking, SEC22B appears to mediate membrane expansion under several conditions, including *Legionella* and *Leishmania* infection in macrophages^9–11^ as well as during axonal growth from isolated cortical neurons^8^. Some evidence suggests that SEC22B contributes to cellular homeostasis as well. For example, in murine macrophages, SEC22B negatively regulates phagocytosis^12^ but promotes reactive oxygen species accumulation during *S*. *aureus* infection^13^. In flies, Sec22 influences ER morphology^14^, while in yeast, Sec22 contributes to autophagosome biogenesis^15^. In human cell lines, SEC22B has been implicated in the secretory autophagy pathway^16^ as well as in macroautophagy^17^. SEC22B also contributes to other secretory pathways, such as that in VLDL (very-low-density lipoprotein)-secreting rat hepatocytes. Thus, current evidence suggests that *Sec22b* is highly conserved and plays a fundamental role in cell biology. However, while cDNA library-based expression profiling has demonstrated that *Sec22b* is expressed in murine embryos^18–20^, its function in embryogenesis *in vivo* remains unexplored.

Utilizing *Cre-Lox* mice, we deleted *Sec22b* from all tissues, from hematopoietic and endothelial cells, and from *CD11c-*expressing cells, a subset of the hematopoietic cell population. We observed that *Sec22b* is critical for embryonic development. Embryos with a global deficiency in *Sec22b* do not survive beyond 8.5 days post coitum (E8.5). Furthermore, deletion of *Sec22b* from the hematopoietic compartment with *Vav1-Cre* results in embryonic lethality. However, normal development was observed with deletion of *Sec22b* in *CD11c*-expressing hematopoietic cells.

## Results

### *Sec22b* is necessary for embryonic development

**Figure 1:**
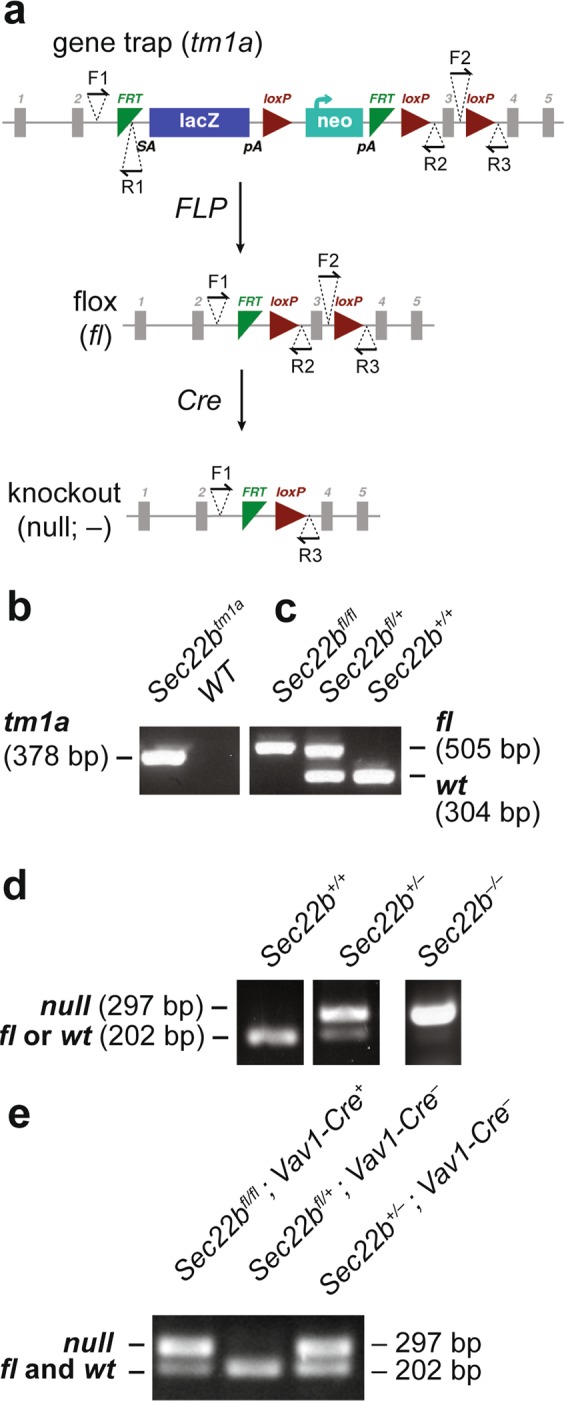
Generation of gene targeted *Sec22b* alleles. (**a**) *Sec22b*-conditional gene trapped mice (*tm1a*), with a FRT-flanked gene trap inserted between exons 2 and 3 were mated to FLP-recombinase transgenic mice to create floxed (*fl*) mice, with *LoxP* sites flanking exon 3. Floxed mice were mated to *EIIa-Cre* to generate the germline null allele (*Sec22b*^−^), or to *Vav1-Cre* or *CD11c-Cre* transgenic mice to generate tissue specific *Sec22b* deficiency. Binding sites for genotyping primers (F1, F2, R1, R2, R3) are indicated with half arrowheads. (**b**) PCR with primers F1 and R1 detects the insertion of the conditional gene trap in *Sec22b* (*tm1a*). (**c**) PCR with primers F1 and R2 detects the excision of the conditional gene trap by FLP recombinase and distinguishes between *Sec22b*^*fl*^ homozygous and heterozygous mice. (**d**) Competitive PCR with primers F1, F2, and R3 detects the excision of exon 3 of *Sec22b* and distinguishes between *Sec22b*^−^ heterozygous and homozygous mice. (**e**) PCR on genomic DNA isolated from peripheral blood of a surviving *Sec22b*^*fl*/*fl*^*; Vav1-Cre*^+^ mouse and a *Sec22b*^*fl*/+^*; Vav1-Cre*^−^ littermate control, compared to DNA from *Sec22b*^+/−^ mice. Gel images (**b**–**e**) are cropped. Full-length images may be found in Supplementary Fig. S1a–c.

To determine the role of SEC22B *in vivo*, we intercrossed mice heterozygous for a FRT recombination site-flanked conditional gene-trapped *Sec22b* allele (*Sec22b*^*tm1a*/+^) (Fig. 1a,b), but did not detect any *Sec22b*^*tm1a*/*tm1a*^ offspring at weaning (*p* < 0.0001) (Table 1). To exclude the possibility that an off target gene trap effect^21^, as opposed to the loss of functional *Sec22b*, was responsible for this phenotype, we generated mice heterozygous for the *Sec22b* null allele (*Sec22b*^+/−^). First, we crossed the *Sec22b*^*tm1a*^ allele to mice expressing FLP recombinase driven by the human β-actin promoter, and excised the gene trap cassette, resulting in the *Sec22b*^*fl*^ allele (Fig. 1a,c), where exon 3 is flanked by *LoxP* sites (Fig. 1a). Subsequently, the *Sec22b*^*fl*^ allele was crossed to mice expressing *Cre* recombinase driven by the germline-expressed *EIIa* promoter, producing the *Sec22b*^−^ allele (Fig. 1a,d). *Sec22b*^+/−^ mice exhibited normal survival (*p* = 0.6473) (Table 1). However, no *Sec22b*^−/−^ pups were observed at weaning (*p* = 0.0008) (Table 1).

**Table 1:** Genotypic distribution of offspring from *Sec22b*^*tm1a*/+^ and *Sec22b*^+/−^ mating schemes.

a. Genotype:	*Sec22b* ^+/+^	*Sec22b* ^*tm1a*/+^	*Sec22b* ^*tm1a*/ *tm1a*^	*p*-value
***Sec22b*** ^***tm1a*/+**^ **×** ***Sec22b*** ^***tm1a*/+**^ **Expected Ratios**	**25%**	**50%**	**25%**	
At weaning (n = 73)	33% (24)	67% (49)	0% (0)	<*0*.*0001*
**b**. **Genotype:**	**Sec22b** ^**+/+**^	**Sec22b** ^**+/−**^		**p-value**
***Sec22b*** ^**+/**−^ **×** ***Sec22b*** ^**+/+**^ **Expected Ratios**	**50%**	**50%**		
Weaning (n = 234)	48% (113)	52% (121)		0.3237
**c**. **Genotype:**	***Sec22b*** ^**+/+**^	***Sec22b*** ^**+/−**^	***Sec22b*** ^**−/−**^	***p*** **-value**
***Sec22b*** ^**+/**−^ **×** ***Sec22b*** ^**+/**−^ **Expected Ratios**	**25%**	**50%**	**25%**	
Weaning (n = 34)	24% (8)	76% (26)	*0%* (*0*)	<*0*.*0001*
E13.5 (n = 9)	22% (2)	78% (7)	0% (0)	0.0751
E11.5 (n = 21)	24% (5)	76% (16)	0% (0)	*0*.*0024*
E9.5 (n = 20)	25% (5)	75% (15)	0% (0)	*0*.*0032*
E8.5 (n = 35)	34% (12)	60% (21)	6% (2)	*0*.*0033*
E7.5 (n = 23)	13% (3)	65% (15)	22% (5)	0.4685
E3.5 (n = 33)	27% (9)	52% (17)	21% (7)	0.3938

(a) Genotypic distribution of offspring at weaning from *Sec22b*^*tm1a*/+^ intercrosses with expected Mendelian distribution. (b) Genotypic distribution of offspring at weaning from *Sec22b*^+/−^ × *Sec22b*^+/+^ crosses compared to expected Mendelian distribution. (c) Genotypic distribution of offspring at weaning and at indicated days post coitum (e.g. E13.5) from *Sec22b*^+/−^ intercrosses as compared to expected Mendelian distribution. *P-*values are calculated from a one-tailed binomial test for (a, c) *Sec22b*^−/−^ and for (b) *Sec22b*^+/−^ versus all other genotypes.

### *Sec22b*^−/−^ mice do not survive beyond E8.5

To determine the stage at which germline loss of *Sec22b* results in embryonic death, we next performed timed matings on *Sec22b*^+/−^ intercrosses. Offspring from this intercross exhibited Mendelian genotypic distribution at E3.5 (*p* = 0.6929) and E7.5 (*p* = 0.4685) (Table 1). However, at E8.5, *Sec22b*^−/−^ mice were significantly underrepresented (*p* = 0.0055) (Table 1). Thereafter, at E9.5, E11.5, and E13.5, no *Sec22b*^−/−^ embryos were observed (Table 1). Thus, *Sec22b*^−/−^ embryos do not survive beyond E8.5.

### Loss of *Sec22b* does not impact embryo size at E7.5

**Figure 2:**
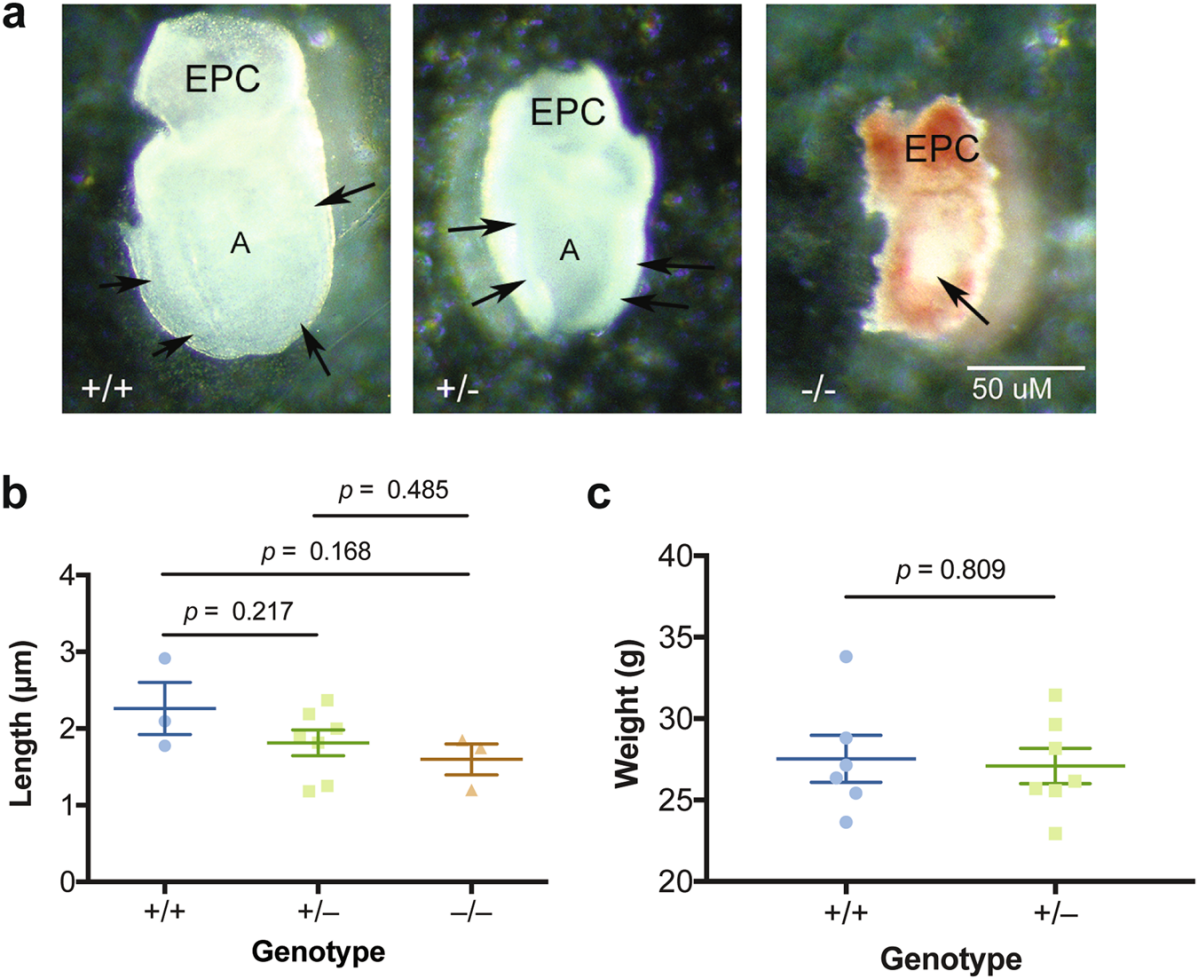
Characterization of *Sec22b*^+/−^ and *Sec22b*^−/−^ mice. (**a**) Lateral views of embryos from the three genotypes. The ectoplacental cone (EPC) is located at the dorsal surface of the embryos. The +/+ (*Sec22b*^+/+^) embryo is beginning to convert to a primitive streak staged embryo, and is characterized by a well defined ectoderm (arrows) surrounding the expanded amniotic cavity, while the +/− (*Sec22b*^+/−^) embryo is at the late egg cylinder stage with a clear ectoderm layer and expanding amniotic cavity. The −/− (*Sec22b*^−/−^) embryo is surrounded by decidua and Reichert’s membrane, the ectoderm is just beginning to elongate to form the egg cylinder (arrow). (**b**) Length (μm) comparison between *Sec22b*^+/+^ (WT, *n* = 3), *Sec22b*^+/−^ (Het, *n* = 7), *Sec22b*^−/−^ (KO, *n* = 3) embryos using an unpaired two-tailed t test. Error bars represent SEM. (**c**) Weight in grams of 4.5–5 month old *Sec22b*^+/−^ (n = 7) and *Sec22b*^+/+^ littermate controls (n = 6) mice using an unpaired two-tailed t test. Error bars represent SEM.

To attempt to investigate the mechanism driving the lethality we observed at E8.5 of *Sec22b*^−/−^ embryos, we examined *Sec22b*^−/−^ embryos one day earlier, at E7.5. At this stage, *Sec22b*^+/−^ and *Sec22b*^−/−^ embryos appeared smaller than *Sec22b*^+/+^ ones (Fig. 2a), though this trend did not reach statistical significance (Fig. 2b). Notably, some *Sec22b*^−/−^ embryos showed a developmental delay, appearing to be in the egg cylinder stage as opposed to the early somite stage (Fig. 2a).

We hypothesized the impact of *Sec22b* heterozygosity on size required additional time to reach significance. Because *Sec22b*^−/−^ mice die *in utero*, to address this, we compared weight of *Sec22b*^+/−^ adult mice to littermate control wildtype mice. However, we did not observe a difference in size, suggesting that *Sec22b* heterozygosity does not impact growth (Fig. 2c).

### *Vav1-Cre* driven deficiency of *Sec22b* results in embryonic lethality

SEC22B has a known role in immune cell function, particularly in mediating intracellular transport in myeloid cells^2,9–13,16^. Interestingly, defects in intracellular transport are known to cause lysosomal storage disorders in humans, which result in early mortality and notably can be treated with bone marrow transplantation^22,23^. Thus, we hypothesized that loss of *Sec22b* in the hematopoietic compartment would lead to embryonic lethality. To test this, we used *Vav1-Cre* to drive deletion of *Sec22b* in hematopoietic tissue. While *Vav1-Cre* also exhibits variable activity between 8–70% in endothelial cells, depending on anatomical location^24^, its excisional efficiency in endothelial tissue remains lower than that in hematopoietic cells^25^.

**Figure 3:**
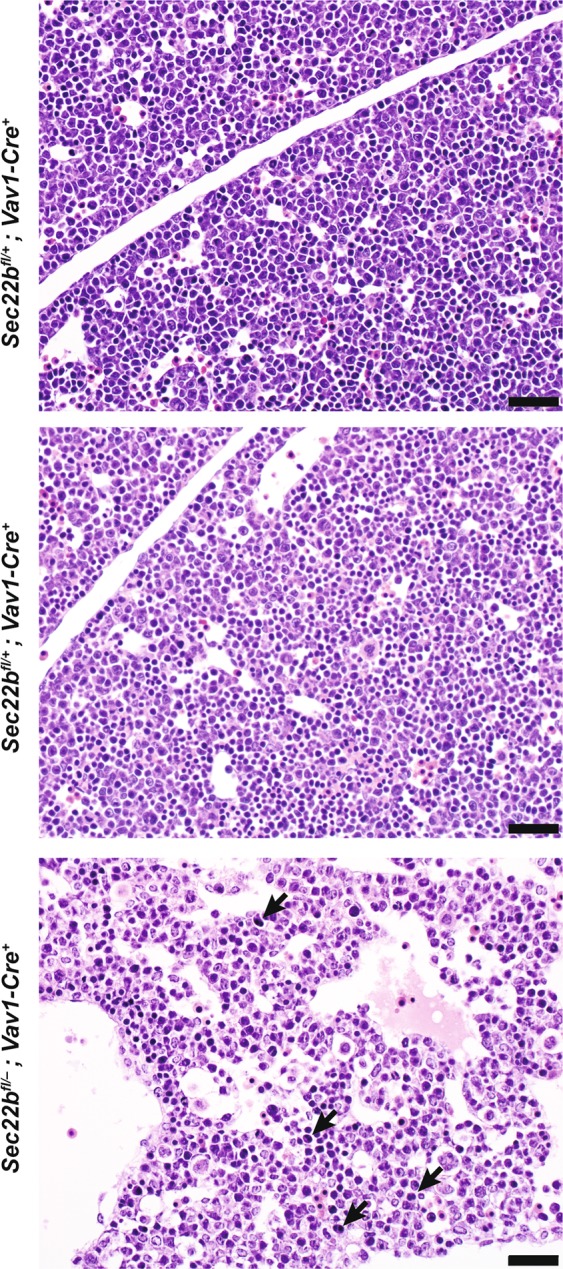
Histopathologic characterization of *Vav1-Cre* mediated excision at E12.5. H&E visualization of E12.5 liver from *Sec22b*^*fl*/−^ *Vav1-Cre*^+^ and *Sec22b*^*fl*/+^ *Vav1-Cre*^−^ embryos with arrows indicating binucleate erythroid progenitors. Size bars all represent 20 μm.

Using a *Sec22b*^*fl*/+^*; Vav1-Cre*^+^ × *Sec22b*^*fl*/*fl*^*; Vav1-Cre*^−^ breeding scheme, we observed a significantly reduced number of *Sec22b*^*fl*/*fl*^*; Vav1-Cre* mice at weaning (*p* < 0.0001) (Table 2). Additionally, we observed a trend towards fewer *Sec22b*^*fl*/*fl*^*; Vav1-Cre*^+^ and *Sec22b*^*fl*/−^*; Vav1-Cre*^+^ embryos at E12.5 (Table 2), one day after *Vav1* expression begins^26,27^. To understand how loss of *Sec22b* in hematopoietic cells and endothelial cells might be causing embryonic lethality, we examined an E12.5 *Sec22b*^*fl*/−^ *Vav1-Cre*^+^ embryo for evidence of dysfunction in hematopoietic organs where hematopoietic stem cells are found. Interestingly, in the liver, we observed enlarged endothelial-lined hepatic sinusoids and binucleate erythroid progenitors (Fig. 3).

**Table 2:** Genotypic distribution of offspring from *Sec22b*^*fl*/+^*; Vav1-Cre*^+^ × *Sec22b*^*fl*/*fl*^*; Vav1-Cre*^−^ mating pairs.

a. Genotype:	*Sec22b* ^*fl*/ *fl*^ *; Vav1-Cre* ^−^	*Sec22b* ^*fl*/+^ *; Vav1-Cre* ^−^	*Sec22b* ^*fl*/+^ *; Vav1-Cre* ^+^	*Sec22b* ^*fl*/ *fl*^ *; Vav1-Cre* ^+^	*p*-value
***Sec22b***^***fl*/+**^***; Vav1-Cre***^**+**^ × ***Sec22b***^***fl*/*fl***^***; Vav1-Cre***^−^ **Expected Ratios**	**25%**	**25%**	**25%**	**25%**	
Observed at weaning (n = 177)	29% (52)	29% (52)	36% (64)	5% (9)	<*0*.*0001*
**b**. **Genotype:**	***Sec22b*** ^***fl*/*fl***^ ***; Vav1-Cre*** ^**+**^	***Sec22b*** ^***fl*/**−^ ***; Vav1-Cre*** ^**+**^	***All other genotypes***		
**1**. ***Sec22b***^***fl*/+**^***; Vav1-Cre***^**+**^ × ***Sec22b***^***fl*/**−^***; Vav1-Cre***^−^ **Expected Ratios**	**12**.**5%**	**12**.**5%**	**75%**		
E12.5 (n = 18)	0% (0)	11.1% (2)	88.9% (16)		0.1353
**2**. ***Sec22b***^**+/**−^***; Vav1-Cre***^**+**^ × ***Sec22b***^***fl*/*fl***^***; Vav1-Cre***^−^ **Expected Ratios**	**12**.**5%**	**12**.**5%**	**75%**		
E12.5 (n = 19)	0% (0)	3.0% (1)	97.0% (18)		*0*.*0310*

(a) Genotypic distribution of offspring at weaning from *Sec22b*^*fl*/+^*; Vav1-Cre*^+^ × *Sec22b*^*fl*/*fl*^*; Vav1-Cre*^−^ crosses as compared to expected Mendelian distribution. (b) Genotypic distribution of offspring at E12.5 from (1) *Sec22b*^*fl*/+^*; Vav1-Cre*^+^ × *Sec22b*^*fl*/−^*; Vav1-Cre*^−^ and (2) *Sec22b*^+/−^*; Vav1-Cre*^+^ × *Sec22b*^*fl*/*fl*^*; Vav1-Cre*^−^ crosses as compared to expected Mendelian distribution. *P-*values for (a) are calculated from a one-tailed binomial test for *Sec22b*^*fl*/*fl*^*; Vav1-Cre*^+^ versus all other genotypes. *P-*values for (b) are calculated from a one-tailed binomial test comparing the knockout embryos (both *Sec22b*^*fl*/*fl*^*; Vav1-Cre*^+^ and *Sec22b*^*fl*/−^*; Vav1-Cre*^+^) versus all other genotypes.

**Figure 4:**
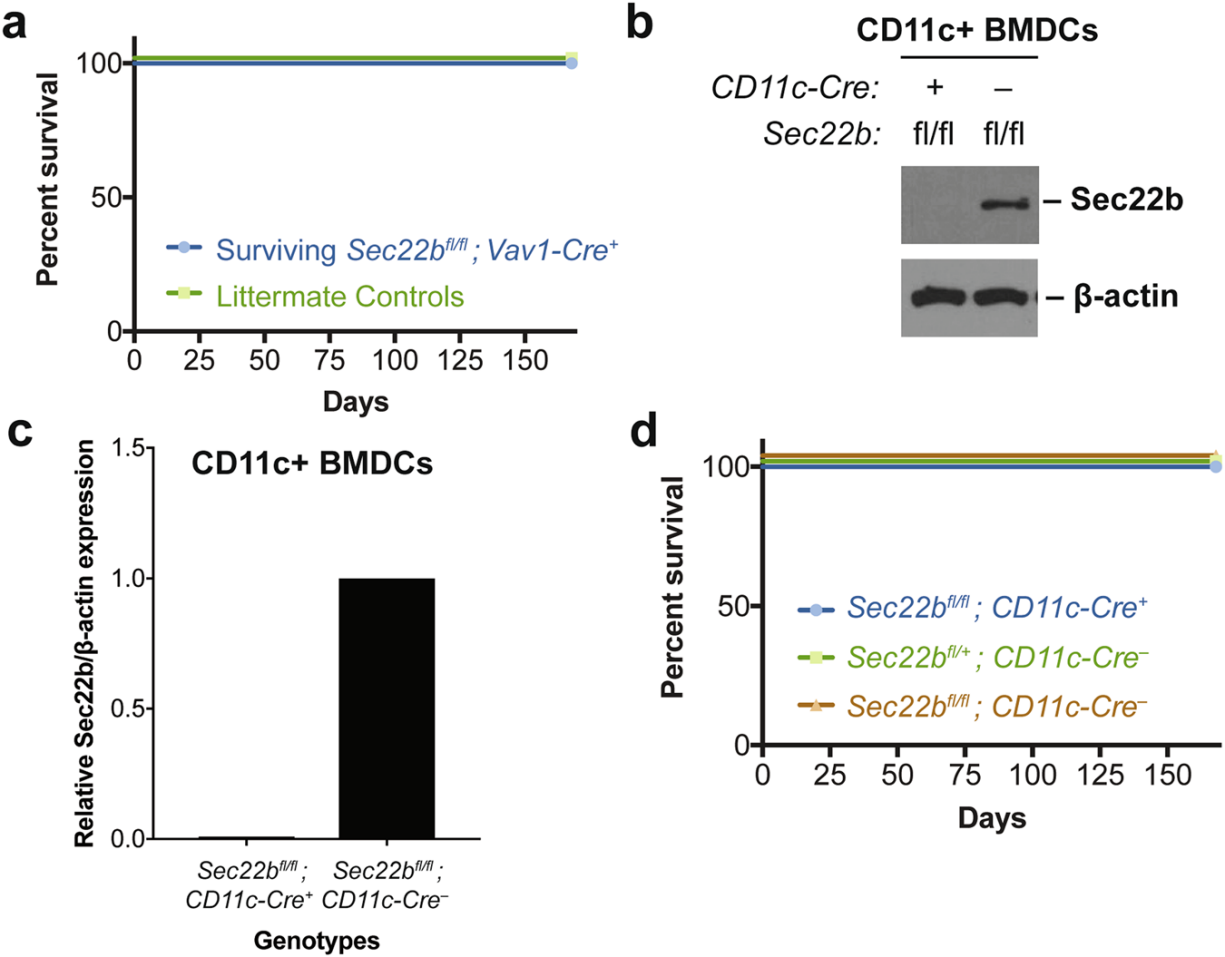
Survival of mice with *Sec22b* deletion in hematopoietic subsets. (**a**) 6 month survival curve for surviving *Sec22b*^*fl*/*fl*^*; Vav1-Cre*^+^ mice (n = 5) as compared to littermates (n = 4). (**b**) Western Blot and (**c**) corresponding quantification of CD11c + MACS-sorted BMDCs from *Sec22b*^*fl*/*fl*^*; CD11c-Cre*^+^ mice and *Sec22b*^*fl*/*fl*^*; CD11c-Cre*^−^. The Western Blot image (**b**) is cropped. Full-length images may be found in Supplementary Fig. S1d. (**d**) 6 month survival curve for *Sec22b*^*fl*/*fl*^*; CD11c-Cre*^+^ mice (n = 53) as compared to *Sec22b*^*fl*/+^*; CD11c-Cre*^−^ (n = 52) and *Sec22b*^*fl*/*fl*^*; CD11c-Cre*^−^ (n = 38) littermates.

Finally, while some *Vav1-Cre*-mediated knockout embryos survived to weaning (Table 2), amplification of genomic DNA at the *Sec22b* locus in peripheral blood cells from these survivors (Fig. 1e) suggests that incomplete excision at exon 3 of *Sec22b* may explain survival to weaning and into adulthood (Fig. 4a).

### *Sec22b*^+/−^ mice exhibit no hematopoietic phenotype under physiologic conditions

**Figure 5:**
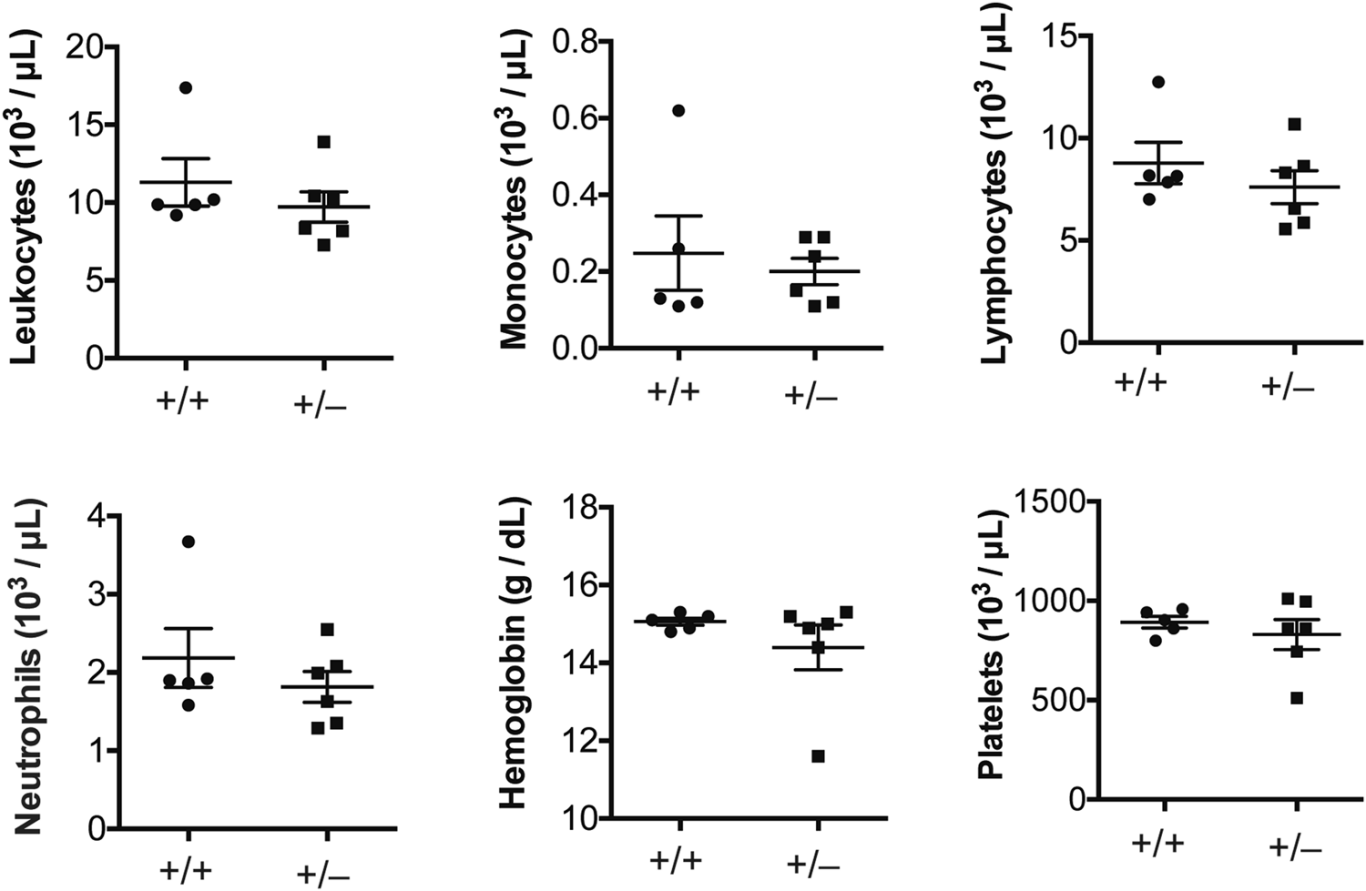
Complete blood counts on *Sec22b* heterozygous mice. Total leukocytes, monocytes, lymphocytes, neutrophils, hemoglobin, and platelets from peripheral blood of 5 month old *Sec22b*^+/+^ (n = 5) and *Sec22b*^+/−^ (n = 6) littermates quantified as indicated on axes compared using unpaired two-tailed t tests. Error bars represent SEM.

Because hematopoietic loss of *Sec22b* results in embryonic lethality (Table 2), we wondered if *Sec22b*^+/−^ mice might exhibit a hematopoietic phenotype. To test this, we performed complete blood counts (CBCs) on peripheral blood collected from *Sec22b*^+/−^ mice. These were indistinguishable from that obtained from littermate controls, including total white blood cells, monocytes, lymphocytes, neutrophils, hemoglobin, and platelet counts (Fig. 5).

### Mice with *Sec22b* deletion in CD11c^+^ cells survive to adulthood

Given the embryonic lethality observed with *Vav1-*mediated deletion of *Sec22b*, the known role for SEC22B in macrophages^10–13^ and dendritic cells (DCs)^2^ *in vitro*, and the role of intracellular transport in lysosomal storage disorders^23^, we hypothesized loss of *Sec22b* in a myeloid cell population would result in embryonic lethality. To test this, we used *Itgax-Cre* (*CD11c-Cre*) to delete *Sec22b* in CD11c-expressing bone-marrow derived DCs (BMDCs) (*Sec22b*^*fl*/*fl*^*; CD11c-Cre*^+^) (Fig. 4b,c). To our surprise, offspring generated by crossing *Sec22b*^*fl*/+^*; CD11c-Cre*^+^ and *Sec22b*^*fl*/*fl*^*;* CD11c-Cre^−^ mice exhibited the expected Mendelian distribution (Table 3) as well as normal survival up to 6 months (168 days) (Fig. 4d).

**Table 3:** Genotypic distribution of offspring from *Sec22b*^*fl*/+^*; CD11c-Cre*^+^ × *Sec22b*^*fl*/*fl*^*; CD11c-Cre*^−^ mating pairs.

Genotype:	*Sec22b* ^*fl*/ *fl*^ *; CD11c-Cre* ^−^	*Sec22b* ^*fl*/+^ *; CD11c-Cre* ^−^	*Sec22b* ^*fl*/+^ *; CD11c-Cre* ^+^	*Sec22b* ^*fl*/ *fl*^ *; CD11c-Cre* ^+^	*p*-value
***Sec22b***^***fl*/+**^***; CD11c-Cre***^**+**^ × ***Sec22b***^***fl*/*fl***^***; CD11c-Cre***^−^**Expected Ratios**	**25%**	**25%**	**25%**	**25%**	
Observed at weaning (n = 606)	28% (171)	25% (149)	22% (133)	25% (153)	0.4596

*G*enotypic distribution of offspring at weaning from *Sec22b*^*fl*/+^*; CD11c-Cre*^+^ × *Sec22b*^*fl*/*fl*^*; CD11c-Cre*^−^ mating pairs as compared to expected Mendelian distribution. *P-*values are calculated from a one-tailed binomial test for *Sec22b*^*fl*/*fl*^*; CD11c-Cre*^+^ versus all other genotypes.

## Discussion

Our studies demonstrate that *Sec22b* is required *in vivo* for survival past E8.5 (Table 1). While we observed normal development of *Sec22b* heterozygous adult mice (Fig. 2c), some null embryos demonstrated a developmental delay at E7.5 (Fig. 2a,b), though this did not reach statistical significance when assessed by embryonic length (Fig. 2b). Thus, the mechanism by which *Sec22b* is required for *in vivo* survival remains undefined.

Our data additionally demonstrate that deletion of *Sec22b* from the hematopoietic compartment also results in partial embryonic lethality (Table 2) and may cause abnormal erythropoiesis, based on preliminary histologic data (Fig. 3a). Further work is necessary to characterize the impact of *Sec22b* on embryonic hematopoiesis. Notably, *Sec22b* heterozygosity does not cause a hematopoietic phenotype under physiologic conditions (Fig. 5) and deletion of *Sec22b* within a specific hematopoietic subpopulation, CD11c-expressing cells, did not reproduce the embryonic lethality (Table 3, Fig. 4d). Interestingly, loss of *Sec22b* in this compartment does not seem to affect the development or function of CD11c^+^ cells^28^.

*Vav1-Cre* also exhibits recombination in endothelial cells, albeit less efficiently than in hematopoietic cells^24^. Thus it is possible that loss of *Sec22b* in endothelial cells may also drive lethality. Whether this is through their function as blood vessel endothelium or as progenitors of hematopoietic stem cells^29^ must be investigated in the future. The significance of altered hepatic sinusoidal morphology (Fig. 3) remains unclear. Interestingly, a recent study described a patient exhibiting growth delay, intellectual disability, hepatopathy, joint contracture and immunodeficiency with multiple homozygous recessive mutations, including in *Sec22b*^30^.

Taken together, our data suggest *Sec22b* is necessary in at least two cell compartments for embryonic survival: *Vav1*-expressing and non-expressing cells. *Vav1* transcripts are first detectable at E11.5^26,27,31^. By E12.5, there were reduced populations of *Sec22*^*fl*/*fl*^*; Vav1-Cre*^+^ and *Sec22b*^*fl*/−^*; Vav1-Cre*^+^ embryos (Table 2), which reached significance by weaning (Table 2), demonstrating the necessity of *Sec22b* expression in *Vav1*^+^ cells for embryonic survival. Furthermore, given that global deletion of *Sec22b* results in embryonic lethality by E8.5 (Table 1), *Sec22b* expression in another tissue compartment is also necessary for embryonic survival.

This *Vav1-*non-expressing cell compartment in which *Sec22b* expression is required for embryonic survival remains undefined. The loss of *Sec22b*^−/−^ embryos at E8.5 could be due to the absence of *Sec22b* in the first wave of embryonic blood cells, which arise in the fetal yolk sac at E7.25^32^, or in another cell compartment. *Sec22b* has also previously been shown to be required *in vitro* for axonal growth in isolated mouse cortical neurons^8^ and for VLDL secretion in rat hepatocytes^33^, while in *D*. *melanogaster*, loss of *Sec22* resulted in defects in eye development^14^. Furthermore, mutations to a SEC22B partner SNARE, GOSR2, have been associated with progressive myoclonic epilepsy^5^. Finally, SEC22B has also been implicated in pancreatic β-cell proinsulin secreation^34^. Future studies may explore how *Sec22b* expression in these tissues alters embryonic development and survival and may facilitate the development of clinically translatable models.

## Methods

### Mice

Mice with a FRT-flanked conditional gene trap inserted between exons 2 and 3 of *Sec22b* were obtained from the European Conditional Mouse Mutagenesis Program (EUCOMM; *Sec22b*^tm1a(EUCOMM)Wtsi^) and crossed to mice with *FLP* recombinase expressed under the control of human β-actin promoter (005703, The Jackson Laboratory) to create the *Sec22b*^*fl*^ allele. *EIIa-Cre* (003724, The Jackson Laboratory), *Vav1-Cre* (008610, The Jackson Laboratory), and *CD11c-Cre* transgenic mice (008068, The Jackson Laboratory), were bred to *Sec22b*^*fl*/*fl*^ mice to create *Sec22b*^*fl*/*fl*^*; EIIa-Cre*^+^, *Sec22b*^*fl*/*fl*^*; Vav1-Cre*^+^, *Sec22b*^*fl*/*fl*^*; CD11c-Cre*^+^ mice. Mice acquired from Jackson Laboratory had been backcrossed to the C57/BL6 background as described in their catalog. Mice acquired from EUCOMM were generated using the C57/BL6 background. All animals were cared for under regulations reviewed and approved by the University of Michigan Institutional Animal Care and Use Committee, based on University Laboratory Animal Medicine guidelines.

### Timed matings

Breeding pairs were co-housed in the evening. Females were checked for the presence of a vaginal plug the following morning (0.5 days post coitum; E0.5). Those with plugs were tracked and euthanized at the appropriate time point and embryos were dissected from uteri and, where indicated, photographed prior to fixation.

### DNA Isolation

Weaned mice and adult mice were genotyped by digesting tail clips in 200 uL/tail clip DirectPCR Lysis Reagent (Mouse Tail) (Viagen, 102-T) and 4 uL/tail clip Proteinase K (Sigma Aldrich, P4850) at 56 °C overnight followed by denaturing at 95 °C for 1 hour. Genomic DNA from embryos aged E7.5–13.5 and from peripheral blood was obtained using the DNeasy Blood & Tissue Kit (Qiagen, 69504), following manufacturer’s instructions. E3.5 blastocysts were harvested into 1xPBS into PCR tubes (USA Scientific, 1402–2500), frozen at −80 °C, and then thawed. Thawed product was used for genotyping PCR.

### Primers and genotyping

Primers used to genotype *Cre* transgenes and the *Sec22b* allele are collected in Table 4. Binding locations for the *Sec22b* primers are identified in Fig. 1a. The *Sec22b* F1 + R1 primers was used to identify gene-trapped mice and the *Sec22b* F1 + R2 primers was used to identify floxed versus wildtype mice. *Sec22b* F1, F2, R3 were used in a competitive PCR to identify the null allele versus the floxed allele. Genotyping was performed via PCR reaction with GoTaq Green Master Mix (Promega, M7122) according to manufacturer’s recommendations.

**Table 4:** Primers used for *Sec22b* and *Cre* genotyping. *Sec22b* primers include F1 and F2 forward primers and R1, R2, and R3 reverse primers. *Sec22b* primer binding positions are indicated in Fig. 1a. *Cre* primers detect both *EIIa*- and *CD11c-Cre* transgenes. Used together, *Vav1* primers detect the *Vav1-Cre* transgene.

Primer Name	*Sequence* (*5*′*→3*′)
*Sec22b* F1	AAGGGTGGATGGATTCTTCACAC
*Sec22b* F2	TCCTTTTGAATGGAGAAAGCTTC
*Sec22b* R1	TTGGTGGCCTGTCCCTCTCACCTT
*Sec22b* R2	GCAGCTCAGCAGTAAGAACACGTC
*Sec22b* R3	CCTGTGACAGTCTACAGATTGGA
*Cre* F	TTACCGGTCGATGCAACGAGT
*Cre* R	TTCCATGAGTGAACGAACCTGG
*Vav1* F1	AGATGCCAGGACATCAGGAACCTG
*Vav1* R1	ATCAGCCACACCAGACACAGAGATC
*Vav1* F2	CTAGGCCACAGAATTGAAAGATCT
*Vav1 R2*	GTAGGTGGAAATTCTAGCATCATC

Gels were imaged using AlphaImager HP. Resulting images were processed using AutoContrast.

### Imaging and analysis

Embryos were dissected and imaged by light microscopy (Leica, DM IRB). ImageJ was used to calculate the surface area of photographed embryos. Images were prepared with Adobe Photoshop CS6, using the AutoContrast and AutoTone features and by setting the gamma correction to 0.5.

### Histologic methods

E12.5 embryos were embedded in OCT compound and frozen. Sections were then placed on glass slides and stained with hematoxylin and eosin.

### Western blot

Whole cell lysates were obtained and protein concentrations determined by BCA Protein Assay (Thermo Scientific, 23225). Protein was separated by SDS-PAGE gel electrophoresis and transferred to PVDF membrane (Millipore, IPVH00010) using a Bio-Rad semi-dry transfer cell (1703940) (20 V, 1 h). Blots were incubated with SEC22B (1:200, Santa Cruz, 29-F7) and b-Actin (1:1000, abcam, ab8226) primary antibodies overnight at 4 °C. Incubation with secondary anti-mouse antibody conjugated to HRP (1:10,000, Santa Cruz, sc-2005) was performed for 1 hour at room temperature. Bound antibody was revealed using SuperSignal ECL substrate (Thermo Scientific). Conversion to grayscale and densitometric analysis was performed using ImageJ.

### Complete blood counts

Peripheral blood was collected into K2 EDTA-coated Microvette collection tubes (Sarstedt, 16.444.100). CBCs were performed with a HEMAVet950 (Drew Scientific, CT) at the University of Michigan *In Vivo* Animal Core.

### Statistics

All statistical analysis was performed using Graphpad Prism 7. Specific tests are indicated in the respective figure legends.

## Supplementary information

Supplementary Information

## Data Availability

All data generated or analysed during this study are included in this published article or in Supplementary Information.

## References

[1] Chen YA, Scheller RH (2001). SNARE-mediated membrane fusion. Nat Rev Mol Cell Biol.

[2] Cebrian I (2011). *Sec22b* regulates phagosomal maturation and antigen crosspresentation by dendritic cells. Cell.

[3] Zhang T, Wong SH, Tang BL, Xu Y, Hong W (1999). Morphological and functional association of Sec22b/ERS-24 with the pre-Golgi intermediate compartment. Mol Biol Cell.

[4] Xu D, Joglekar AP, Williams AL, Hay JC (2000). Subunit structure of a mammalian ER/Golgi SNARE complex. J Biol Chem.

[5] Volker JM (2017). Functional assays for the assessment of the pathogenicity of variants of GOSR2, an ER-to-Golgi SNARE involved in progressive myoclonus epilepsies. Dis Model Mech.

[6] Garcia-Castillo MD (2015). Retrograde transport is not required for cytosolic translocation of the B-subunit of Shiga toxin. J Cell Sci.

[7] Aoki T, Kojima M, Tani K, Tagaya M (2008). Sec22b-dependent assembly of endoplasmic reticulum Q-SNARE proteins. Biochem J.

[8] Petkovic Maja, Jemaiel Aymen, Daste Frédéric, Specht Christian G., Izeddin Ignacio, Vorkel Daniela, Verbavatz Jean-Marc, Darzacq Xavier, Triller Antoine, Pfenninger Karl H., Tareste David, Jackson Catherine L., Galli Thierry (2014). The SNARE Sec22b has a non-fusogenic function in plasma membrane expansion. Nature Cell Biology.

[9] Arasaki K, Roy CR (2010). Legionella pneumophila promotes functional interactions between plasma membrane syntaxins and Sec22b. Traffic.

[10] Arasaki K, Toomre DK, Roy CR (2012). The Legionella pneumophila effector DrrA is sufficient to stimulate SNARE-dependent membrane fusion. Cell Host Microbe.

[11] Canton J, Ndjamen B, Hatsuzawa K, Kima PE (2012). Disruption of the fusion of Leishmania parasitophorous vacuoles with ER vesicles results in the control of the infection. Cell Microbiol.

[12] Hatsuzawa K (2009). Sec22b is a negative regulator of phagocytosis in macrophages. Mol Biol Cell.

[13] Abuaita BH, Burkholder KM, Boles BR, O’Riordan MX (2015). The Endoplasmic Reticulum Stress Sensor Inositol-Requiring Enzyme 1alpha Augments Bacterial Killing through Sustained Oxidant Production. MBio.

[14] Zhao X (2015). Sec. 22 regulates endoplasmic reticulum morphology but not autophagy and is required for eye development in Drosophila. J Biol Chem.

[15] Nair U (2011). SNARE proteins are required for macroautophagy. Cell.

[16] Kimura T (2017). Dedicated SNAREs and specialized TRIM cargo receptors mediate secretory autophagy. EMBO J.

[17] Renna M (2011). Autophagic substrate clearance requires activity of the syntaxin-5 SNARE complex. J Cell Sci.

[18] Ko MS (2000). Large-scale cDNA analysis reveals phased gene expression patterns during preimplantation mouse development. Development.

[19] Carninci P (2005). The transcriptional landscape of the mammalian genome. Science.

[20] Hay JC, Chao DS, Kuo CS, Scheller RH (1997). Protein Interactions Regulating Vesicle Transport between the Endoplasmic Reticulum and Golgi Apparatus in Mammalian Cells. Cell.

[21] Maguire S (2014). Targeting of Slc25a21 is associated with orofacial defects and otitis media due to disrupted expression of a neighbouring gene. PLoS One.

[22] Hoogerbrugge PM (1995). Allogeneic bone marrow transplantation for lysosomal storage diseases. The European Group for Bone Marrow Transplantation. Lancet.

[23] Platt FM (2014). Sphingolipid lysosomal storage disorders. Nature.

[24] Inra CN (2015). A perisinusoidal niche for extramedullary haematopoiesis in the spleen. Nature.

[25] de Boer J (2003). Transgenic mice with hematopoietic and lymphoid specific expression of Cre. Eur J Immunol.

[26] Zmuidzinas A (1995). The vav proto-oncogene is required early in embryogenesis but not for hematopoietic development *in vitro*. EMBO J.

[27] Bustelo XR, Rubin SD, Suen KL, Carrasco D, Barbacid M (1993). Developmental expression of the vav protooncogene. Cell Growth Differ.

[28] Wu SJ (2017). A Critical Analysis of the Role of SNARE Protein SEC22B in Antigen Cross-Presentation. Cell Rep.

[29] Eilken HM, Nishikawa S, Schroeder T (2009). Continuous single-cell imaging of blood generation from haemogenic endothelium. Nature.

[30] Diao H, Zhu P, Dai Y, Chen W (2018). Identification of 11 potentially relevant gene mutations involved in growth retardation, intellectual disability, joint contracture, and hepatopathy. Medicine (Baltimore).

[31] Keller G, Kennedy M, Papayannopoulou T, Wiles MV (1993). Hematopoietic commitment during embryonic stem cell differentiation in culture. Mol Cell Biol.

[32] Palis J, Robertson S, Kennedy M, Wall C, Keller G (1999). Development of erythroid and myeloid progenitors in the yolk sac and embryo proper of the mouse. Development.

[33] Siddiqi S, Mani AM, Siddiqi SA (2010). The identification of the SNARE complex required for the fusion of VLDL-transport vesicle with hepatic cis-Golgi. Biochem J.

[34] Fan J (2017). cTAGE5 deletion in pancreatic beta cells impairs proinsulin trafficking and insulin biogenesis in mice. J Cell Biol.

