# Supplementary material for: SNARE protein SEC22B regulates early embryonic development
Source: Sci Rep. 2019 Aug 7;9:11434. doi: 10.1038/s41598-019-46536-7 (PMC6685974; doi:10.1038/s41598-019-46536-7)
Supplement: Supplementary file 1 — Supplementary Information [file 41598_2019_46536_MOESM1_ESM.docx]

**Supplementary Information**

**SNARE protein SEC22B regulates early embryonic development**

Shin-Rong J Wu^1,2^, Rami Khoriaty^3^, Stephanie H. Kim^1,2^, K Sue O’Shea^4^, Guojing Zhu^5^, Mark Hoenerhoff^6^, Cynthia Zajac^3^, Katherine Oravecz-Wilson^3^, Tomomi Toubai^3^, Yaping Sun^3^, David Ginsburg^3,5,7,8^, Pavan Reddy^1,3*^

^1^Program in Immunology, University of Michigan Medical School, Ann Arbor, USA

^2^Medical Scientist Training Program, University of Michigan Medical School, Ann Arbor

^3^Department of Internal Medicine, Michigan Medicine, Ann Arbor, USA

^4^Department of Cellular and Developmental Biology, University of Michigan Medical School, Ann Arbor, USA

^5^Life Sciences Institute, University of Michigan, Ann Arbor, USA

^6^Unit for Laboratory Animal Medicine, University of Michigan, Ann Arbor, USA

^7^Department of Human Genetics, University of Michigan Medical School, Ann Arbor, USA

^8^Howard Hughes Medical Institute, University of Michigan, Ann Arbor, USA

Lead contact: Pavan Reddy

E-mail: [reddypr@med.umich.edu](mailto:reddypr@med.umich.edu)

Phone: 734-936-7507

**
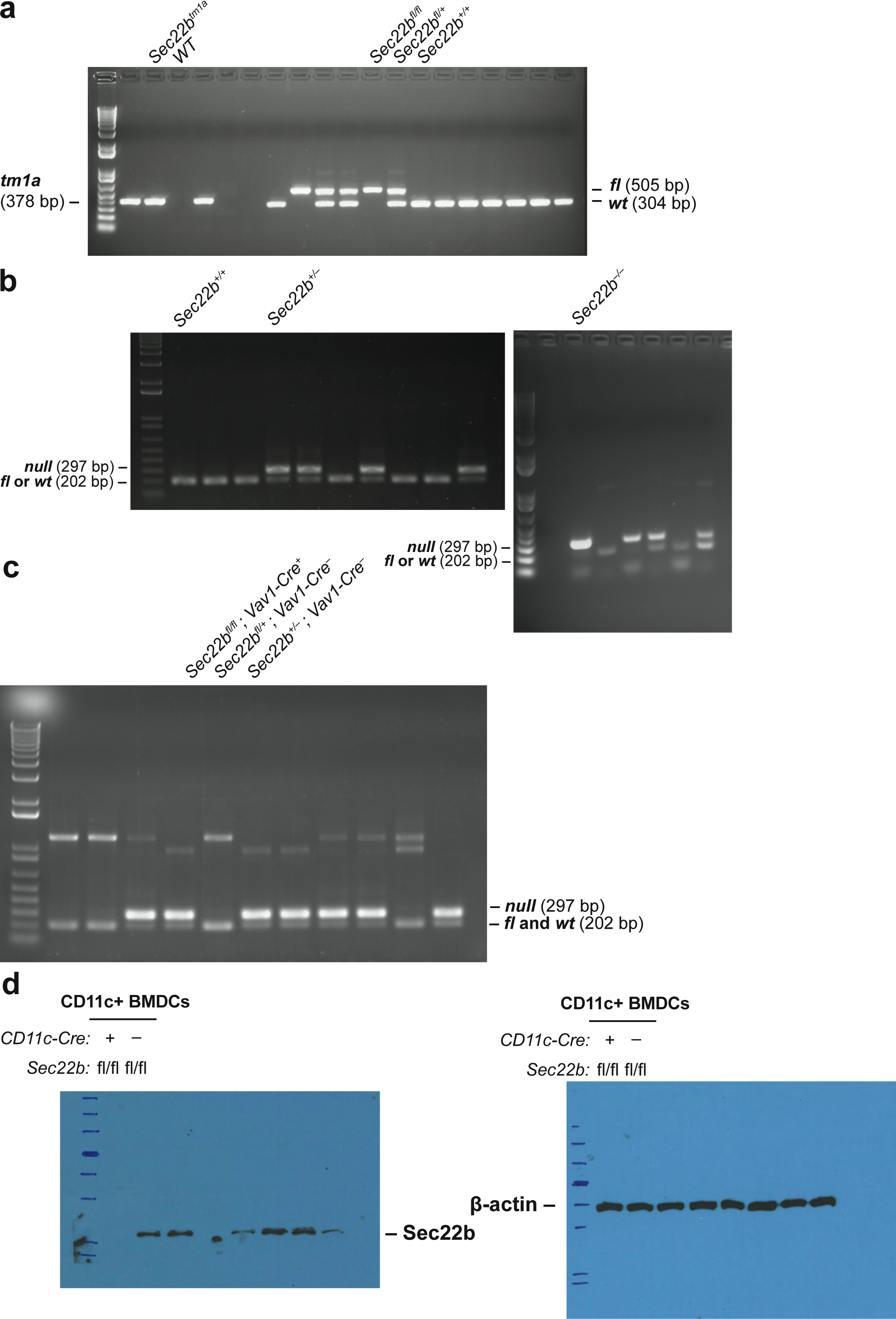
**

**Supplementary Figure S1. (a)** Full-length gel from Figures 1b and 1c. **(b)** Full-length gel from Figure 1d. **(c)** Full-length gel from Figure 1e. **(d)** Full-length Western Blot from Figure 4b.
